# Perindopril/Ambrosin Combination Mitigates Dextran Sulfate Sodium-Induced Colitis in Mice: Crosstalk between Toll-Like Receptor 4, the Pro-Inflammatory Pathways, and SIRT1/PPAR-γ Signaling

**DOI:** 10.3390/ph15050600

**Published:** 2022-05-13

**Authors:** Ahmed M. Kabel, Aliaa Atef, Hany M. Borg, Azza A. K. El-Sheikh, Hana J. Al Khabbaz, Hany H. Arab, Remon S. Estfanous

**Affiliations:** 1 Department of Pharmacology, Faculty of Medicine, Tanta University, Tanta 31527, Egypt; 2 Department of Pathology, Faculty of Medicine, Tanta University, Tanta 31527, Egypt; aliaa.shamseldeen@med.tanta.edu.eg; 3 Physiology Department, Faculty of Medicine, Kafrelsheikh University, Kafr El-Shaikh 33516, Egypt; kemo162008@gmail.com; 4 Basic Health Sciences Department, College of Medicine, Princess Nourah bint Abdulrahman University, P.O. Box 84428, Riyadh 11671, Saudi Arabia; aaelsheikh@pnu.edu.sa; 5 Biochemistry Division, College of Pharmacy, Riyadh Elm University, Riyadh 11681, Saudi Arabia; hanabio@riyadh.edu.sa; 6 Department of Pharmacology and Toxicology, College of Pharmacy, Taif University, P.O. Box 11099, Taif 21944, Saudi Arabia; h.arab@tu.edu.sa; 7 Anatomy and Embryology Department, Faculty of Medicine, Tanta University, Tanta 31527, Egypt; remon.estfanous@med.tanta.edu.eg

**Keywords:** perindopril, ambrosin, dextran sulfate sodium, inflammation, colitis, mice

## Abstract

Colitis is one of the inflammatory states that affect the intestinal wall and may even predispose to malignancy due to chronic irritation. Although the etiology of colitis is not yet fully explored, a combination of genetic and environmental factors is strongly incriminated. Perindopril is an angiotensin-converting enzyme inhibitor that is used for the management of a wide range of cardiovascular diseases. Ambrosin is a sesquiterpene lactone that was proven to have beneficial effects in disorders characterized by inflammatory nature. The objective of this study is to make a comparison between the effects of perindopril or ambrosin on dextran sulfate sodium (DSS)-induced colitis in mice and to explore the effect of their combination. The present findings indicate that each ambrosin or perindopril alone or in combination is able to ameliorate oxidative stress and suppress the proinflammatory pathways in the colonic tissues of DSS-treated mice via mechanisms related to toll-like receptor 4/nuclear factor kappa B signaling and modulation of peroxisome proliferator-activated receptor gamma/sirtuin-1 levels. In addition, each ambrosin or perindopril alone or in combination inhibits apoptosis and augments the mediators of autophagy in DSS-treated mice. These effects are reflected in the amelioration of the histopathological and electron microscopic changes in the colonic tissues. Interestingly, the most remarkable effects are those encountered with the perindopril/ambrosin combination compared to the groups treated with each of these agents alone. In conclusion, the perindopril/ambrosin combination might represent an effective modality for mitigation of the pathogenic events and the clinical sequelae of colitis.

## 1. Introduction

Colitis is a group of characteristic features that signify a form of inflammatory bowel disease [1]. The pathognomonic signs of colitis include the formation of characteristic ulcers or open sores that may lead to diffuse scarring of the colonic tissues [2]. In spite of the advancement in diagnostic techniques in the last decades, the etiology of this condition is still not completely elucidated [3]. However, recent studies concluded that colitis is a genetically determined autoimmune inflammatory disease elicited by certain factors related to environmental and lifestyle conditions [4,5]. The clinical presentation of colitis often involves intermittent attacks of diarrhea with or without blood, with symptom-free periods in between [6]. Interestingly, colitis may be misdiagnosed with other diseases of the colon and may require invasive maneuvers such as endoscopic examination to verify the diagnosis [7]. Up till now, there has been no remedy to induce a complete cure of colitis, although agents with anti-inflammatory effects represent the most frequently prescribed medications for this condition [8].

A wide range of inflammatory pathways have been proven to play a fundamental role in the initiating events and the sequelae of colitis [9]. For example, toll-like receptor 4 (TLR-4) was reported to be overexpressed in cases of inflammatory bowel disease and was concomitantly associated with increased tissue levels of nuclear factor kappa B (NF-κB), which is considered the key mediator of the inflammatory process [10]. In addition, TLR-4 has been proven to activate the NLRP3 inflammasome signaling pathway, which represents the cornerstone of the pathophysiology of inflammatory bowel diseases [11]. Recent reports suggested that NLRP3 inflammasome augments colitis via affection of pyroptosis, a new type of proinflammatory programmed cell death, which has many differences from apoptosis and necrosis [12]. Moreover, activation of TLR-4 signaling is associated with increased production of reactive oxygen species (ROS) that elicits a series of secondary changes that ameliorate the antioxidant effects of sirtuin-1 (SIRT-1) in the colonic tissues [13].

Perindopril is an angiotensin-converting enzyme (ACE) inhibitor that has a crucial role in the management of cardiovascular disorders. Recent reports suggest that the renin-angiotensin system may contribute to a large extent to the pathophysiology of inflammatory bowel diseases [14]. Jacobs et al. [15] stated that angiotensin II has pro-inflammatory effects on the colonic tissues, directly via activation of NF-κB and increased tumor necrosis alpha (TNF-α) production from the macrophages and indirectly through increased generation of ROS. Moreover, the levels of angiotensin II were higher in the colonic biopsies obtained from individuals with Crohn’s colitis relative to those obtained from the healthy controls. Interestingly, there was a positive correlation between the colonic mucosal levels of angiotensin II and the degree of macroscopic inflammation in Crohn’s colitis [16]. These reports may confer a role for perindopril in the amelioration of dextran sulfate sodium (DSS)-induced colitis.

Ambrosin is a sesquiterpene lactone that is extracted from *Ambrosia maritima* worldwide [17]. Recent studies have tried to throw light on the potential anti-inflammatory activities of ambrosin that may be of benefit in a wide range of illnesses characterized by an inflammatory nature [18]. These properties may be largely derived from its inhibitory effect on the signaling pathways, in which NF-κB plays a key role [19]. This, in addition to its well-documented antioxidant and antiapoptotic properties, might represent hope for patients with inflammatory bowel diseases [18,20]. The purpose of this study was to make a comparison between the effects of perindopril and ambrosin on DSS-induced colitis in mice and to explore the effect of their combination.

## 2. Methods and Materials

### 2.1. Drugs and Chemicals

DSS was supplied as a powder form by Sigma Aldrich Co., St. Louis, MO, USA (CAS No. 9011-18-1) and was dissolved in distilled water to reach a final concentration of 4%. Perindopril was purchased from Cayman Chemical, Ann Arbor, Michigan, USA (CAS No. 82834-16-0). Ambrosin was obtained from Henan Tianfu Chemical Co., Zhengzhou, China (CAS No. 509-93-3). All other chemicals and reagents were supplied as a kind gift from SimSon Pharma Limited, Maharashtra, India, and were of analytical grade. Both perindopril and ambrosin were suspended in 1.5% carboxymethyl cellulose (CMC) solution.

### 2.2. The Experimental Design

This study was executed according to Helsinki declaration of animal ethics and was approved by the Research Ethics Committee of Faculty of Medicine, Tanta University, Egypt (Approval code 34453/2). Forty-eight Balb/c male mice weighing about 16–26 g were obtained from a local source and kept in metabolic cages at 22–26 °C, 55–62% relative humidity, 12 h light, with free access to food and tap water ad libitum. Mice were randomized into six equal groups each of 8 mice by a laboratory technician who was unaware of the nature of the study as follows: control untreated group; DSS-induced colitis group drank distilled water containing 4% w/v DSS for 7 days [21]; DSS + CMC group received 0.5 mL CMC 1.5% solution by oral gavage; DSS + Perindopril group received perindopril by oral gavage in a dose of 1 mg/kg/day [22]; DSS + Ambrosin group received ambrosin by oral gavage in a dose of 10 mg/kg/day [18]; DSS + Perindopril + Ambrosin group was treated with perindopril concomitantly with ambrosin by oral gavage in the fore-mentioned doses. Treatment with CMC, perindopril, and ambrosin started one week before administration of DSS and continued during the administration of DSS.

### 2.3. Evaluation of Colitis

After DSS administration, mice were examined daily regarding stool consistency, the presence of occult or frank bleeding, and changes in body weight. The data obtained were utilized to calculate the disease activity index (DAI) as indicated in Table 1 according to Friedman et al. [23].

On the 8th day after starting administration of DSS, mice were anesthetized with intraperitoneal injection of thiopental sodium (50 mg/kg). Their abdomens were opened by a midline laparotomy incision, the colon was excised, and its length was measured. Then, the colon was longitudinally opened and washed with saline. A part of the excised colon was homogenized in 5% EDTA/NaCl buffer (pH 4.7). The yielded homogenate was centrifuged at 10,000× *g* for 15 min at 4 °C. The supernatant was separated and utilized for calculation of the biochemical parameters. Other parts of the colon were processed for histopathological, immunohistochemical, and electron microscopic examination. 

### 2.4. Assessment of the Antioxidant Status of the Colonic Tissues

Commercial ELISA kits obtained from MyBioSource, Inc., San Diego, CA, USA (Catalogue No. MBS8579849) were utilized for quantification of ROS in the colonic tissues. Glutathione peroxidase (GPx) and glutathione reductase (GR) were assessed in the colonic tissues using ELISA kits purchased from LSBio, Seattle, WA, USA (Catalogue No. LS-F28917-1 and LS-F24170-1, respectively). The kits adopted the competitive ELISA technique according to the manufacturers’ guide. 

### 2.5. Determination of Nuclear Factor-Erythroid Factor 2-Related Factor 2 (Nrf2), Inducible Nitric Oxide Synthase (iNOS), Peroxisome Proliferator-Activated Receptor Gamma (PPAR-γ), and SIRT-1 in the Colonic Tissues

Nrf2 content of the colonic tissues was assayed using commercial kits supplied by LSBio, Seattle, WA, USA (Catalogue No. LS-F32101-1). Assessment of tissue iNOS was carried out using commercially available kits obtained from BioVision, Waltham, MA, USA (Catalogue No. E4691). ELISA kits purchased from MyBioSource, Inc., San Diego, CA, USA were utilized for assay of the colonic tissues level of PPAR-γ and SIRT-1 levels (Catalogue No. MBS2501353 and MBS2516070, respectively). Sandwich ELISA technique was utilized for assessment of these parameters following the vendor’s instructions. 

### 2.6. Assessment of the Inflammatory Microenvironment and NLR Family Pyrin Domain Containing 3 (NLRP3) Inflammasome in the Colonic Tissues

Tissue interleukin-1 beta (IL-1β), IL-6, and transforming growth factor beta 1 (TGF-β1) were assayed in the colonic tissues using commercial ELISA kits supplied by LSBio, Seattle, WA, USA (Catalogue No. LS-F5626-1, LS-F11810-1, and LS-F5184-1, respectively). Toll-like receptor 4 (TLR-4) content of the colonic tissues was quantified using kits purchased from Abbexa, Cambridge, United Kingdom (Catalogue No. abx570401). ELISA kits of Abcam, USA (Catalogue No. ab279417) were utilized for assessment of the levels of NLRP3 inflammasome in the colonic tissues. Vendor’s guide enclosed with the aforementioned kits was followed for assessment of the inflammatory microenvironment of the colonic tissues.

### 2.7. Determination of c-Fos, c-JUN, and the Phosphorylated Mitogen-Activated Protein Kinase (p38 MAPK) in the Colonic Tissues

Commercially available kits provided by Abbexa, Cambridge, United Kingdom were used for assessment of the protein levels of c-Fos and c-JUN in the colonic tissues (Catalogue No. abx154033 and abx353297, respectively). ELISA kits supplied by MyBioSource, Inc., San Diego, CA, USA (Catalogue No. MBS722905) were used for determination of p38 MAPK (Phospho) in the colonic tissues following the provider’s guide.

### 2.8. Assessment of Colonic Tissue Beclin-1, LC3-II, Caspase-8, and Bcl-2 

Beclin-1 levels were assessed using kits provided by Abbexa, Cambridge, United Kingdom (Catalogue No. abx254844). The levels of LC3-II were quantified using ELISA kits supplied by MyBioSource, Inc., San Diego, CA, USA (Catalogue No. MBS3806182). Caspase-8 and Bcl-2 levels were assayed using commercial ELISA kits purchased from LSBio, Seattle, WA, USA (Catalogue No. LS-F4275-1 and LS-F4136-1, respectively). Sandwich ELISA technique was used for assessment of the aforementioned parameters following the providers’ instructions. 

### 2.9. Evaluation of the Histopathologic Changes of the Colonic Tissues

Specimens of the colon were fixed in 10% neutral buffered formalin, embedded in paraffin, and then cut at 5 µ thickness using a microtome. After deparaffinization, the cut tissue sections were stained with hematoxylin and eosin (H and E) and the severity of colitis was quantified according to the histological disease score of Hirata et al. [24]. Briefly, this score consists of the following five grades: grade 0 denotes normal mucosa of the colon; grade 1 denotes loss of one-third of the mucosal crypts; grade 2 denotes loss of two-thirds of the mucosal crypts; grade 3 involves covering of the lamina propria with a single layer of epithelium associated with mild inflammatory cellular infiltration; grade 4 involves the presence of erosions associated with marked infiltration with inflammatory cells. Eight fields with a magnification of ×100 were randomly selected and examined by a pathologist who was blinded to the nature of the study and the treatment protocol. By determination of the grades of the histopathological changes in the eight fields, the mean values of the histological disease score were calculated.

### 2.10. Assessment of NF-kB (p65) Immunostaining in the Colonic Tissues

Tissue sections were obtained from formalin-fixed paraffin-embedded blocks on positively charged slides and dewaxed with xylene. After that, endogenous peroxidase blocking was performed using 3% hydrogen peroxide slide immersion method. Then, antigen retrieval was carried out using citrate buffer and non-specific staining blocking was performed by normal goat serum. The processed sections were incubated with the primary antibody polyclonal IgG to NF-κB (p65) (Thermo scientific Lab vision, Catalogue No. RB9034-R7) at 1:100 dilution for one hour at room temperature. Then, incubation with the secondary biotinylated antibody was carried out for thirty minutes in humidity chamber followed by exposure to streptavidin enzyme label for thirty minutes. DAB chromogen was utilized as the working coloring agent with counterstaining with Mayer’s hematoxylin stain. NF-κB (p65) was assessed by detecting the activated subunit p65 in the colonic tissues. The percentage of positive nuclear staining was quantified by IHC profiler tool in image analysis software. Evaluation of NF-kB (p65) immunostaining was carried out semi-quantitatively according to both the intensity and quantification of the positively stained nuclei. They were quantified as 0 (negative) refers to no nuclear immunostaining; +1 (weak positive) denoting positive nuclear immunostaining in 1–10% of the cells under examination; +2 (moderately positive) refers to positive nuclear immunostaining in 11–25% of the cells under examination; +3 (strongly positive) denoting positive nuclear immunostaining in over 25% of the cells under examination [25].

### 2.11. Evaluation of the Electron Microscopic Changes of the Colonic Tissues

Samples from the colonic tissues were first washed with PBS, then fixed in 1% osmic acid at 25 °C for 2 h. After that, they were dehydrated by acetone and embedded in Spurr Embedding medium and incubated at 60 °C for 48 h. Then, these sections were cut at 70 nm thickness, stained with uranyl acetate for 20 min and lead citrate for 5 min at 25 °C. These stained sections were examined using JEOL-1010 transmission electron microscope (JEOL, Ltd., Tokyo, Japan).

### 2.12. Statistical Evaluation

Results obtained were statistically evaluated using Minitab software version 18 and were expressed as mean ± standard deviation (S.D.). The normality of the obtained results was checked using the Shapiro–Wilk normality test. The statistical differences between the different groups were evaluated using one-way analysis of normality of variance (ANOVA) followed by Tukey–Kramer multiple comparisons. *p*-value less than 0.05 was considered as the minimally accepted significance level.

## 3. Results

### 3.1. Perindopril and/or Ambrosin Significantly Mitigated the Changes in DAI and Colon Length in DSS-Treated Animals

**Figure 1:**
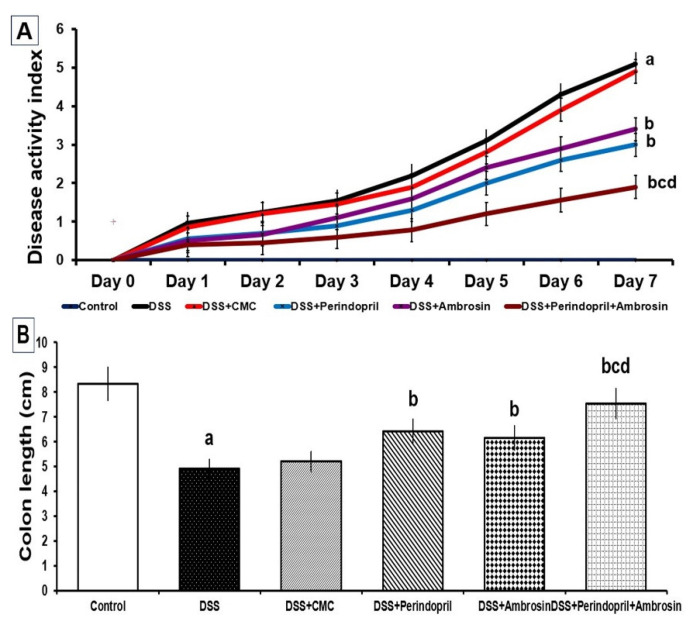
Perindopril with or without ambrosin mitigated the disease activity index (DAI) (**A**) and restored the colon length (**B**) in mice treated with dextran sulfate sodium (DSS) (Mean ± SD); where “a” denotes significant vs. the control group (*p*-value less than 0.05); “b” denotes significant vs. DSS group (*p*-value less than 0.05); “c” denotes significant vs. DSS + perindopril group (*p*-value less than 0.05); “d” denotes significant vs. DSS + ambrosin group (*p*-value less than 0.05).

Administration of DSS to mice elicited a significant decrease in the colon length with a significant elevation of DAI levels relative to the control untreated group. CMC administration to DSS mice did not significantly affect the colon length or DAI levels in comparison to mice treated with DSS alone. DSS mice treated with either ambrosin or perindopril exhibited a significant decline in DAI levels with the restoration of the colon length compared to mice treated with DSS alone. These changes were significant in the perindopril/ambrosin combination group relative to the use of either DSS + perindopril or DSS + ambrosin (Figure 1).

### 3.2. Perindopril and/or Ambrosin Significantly Activated SIRT1/PPAR-γ Signaling in the Colonic Tissues of DSS-Treated Animals

**Figure 2:**
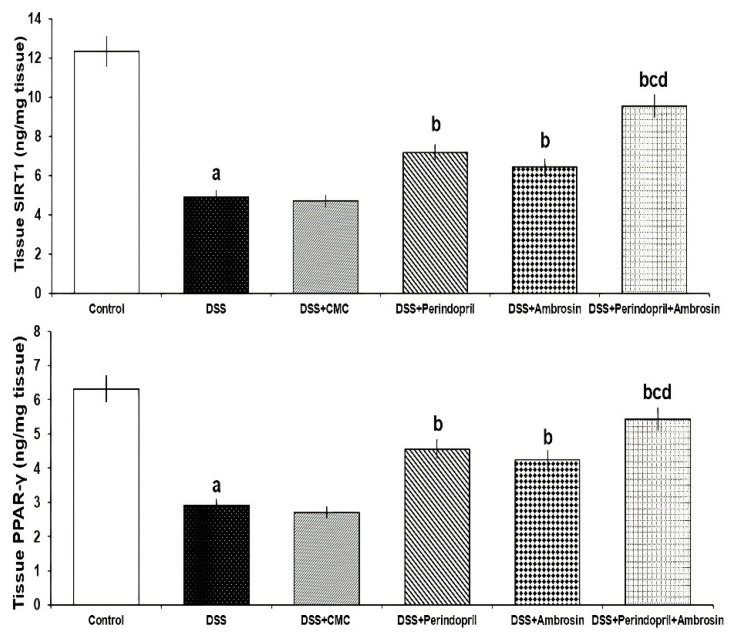
Effect of perindopril with or without ambrosin on SIRT1/PPAR-γ signaling in the colonic tissues of DSS-treated mice (Mean ± SD); where “a” denotes significant vs. the control group (*p*-value less than 0.05); “b” denotes significant vs. DSS group (p-value less than 0.05); “c” denotes significant vs. DSS + perindopril group (*p*-value less than 0.05); “d” denotes significant vs. DSS + ambrosin group (*p*-value less than 0.05).

The significant decline in SIRT1 and PPAR-γ signaling induced by administration of DSS was mitigated with treatment with either perindopril or ambrosin. This mitigating effect was significantly more pronounced in the group treated with the perindopril/ambrosin combination relative to the groups treated with each of these agents alone. The effect of treatment of DSS mice with CMC on SIRT1 and PPAR-γ signaling was insignificant relative to mice treated with DSS alone (Figure 2).

### 3.3. Perindopril and/or Ambrosin Restored the Pro-Oxidant/Antioxidant Balance and iNOS/Nrf2 Signaling in the Colonic Tissues of DSS-Treated Animals

Administration of DSS was associated with a significant decrement in GR, GPx, and Nrf2 content of the colonic tissues, which was associated with a significant increase in ROS and iNOS relative to the control untreated group. These effects were reversed with the administration of either ambrosin or perindopril to DSS-treated mice, but the perindopril/ambrosin combination group attained the upper hand over the use of each of these agents alone. The changes induced in the aforementioned biochemical parameters induced by DSS administration were not affected with the administration of CMC (Table 2).

### 3.4. Perindopril and/or Ambrosin Significantly Decreased the Levels of TLR4, IL-1β, IL-6, and TGF-β1 in the Colonic Tissues of DSS-Treated Animals

Significant augmentation of the levels of TLR4, IL-1β, IL-6, and TGF-β1 was elicited with DSS administration to mice relative to the control group. Interestingly, groups treated with perindopril with or without ambrosin exhibited significant repression of the levels of these parameters, with the maximally detected effect with the use of the perindopril/ambrosin combination relative to administration of each of these agents alone. Administration of CMC to DSS-treated mice did not significantly affect the levels of the aforementioned biochemical parameters when put in comparison with mice treated with DSS alone (Table 3).

### 3.5. Perindopril and/or Ambrosin Mitigated p38 MAPK/c-Fos/c-Jun Pro-Inflammatory Pathways and Suppressed NLRP3 Inflammasome Levels in the Colonic Tissues of DSS-Treated Animals

The significant increase in the levels of p38 MAPK, c-Fos, c-Jun, and NLRP3 inflammasome encountered in the present study in mice treated with DSS alone was combatted with the administration of ambrosin with or without perindopril, with the maximally encountered effect in the group treated with the perindopril/ambrosin combination. However, the administration of CMC did not have a significant influence on the levels of the aforementioned parameters relative to mice treated with DSS alone (Table 4).

### 3.6. Effect of Perindopril and/or Ambrosin on Beclin-1 and LC3-II Levels in the Colonic Tissues of DSS-Treated Animals

**Figure 3:**
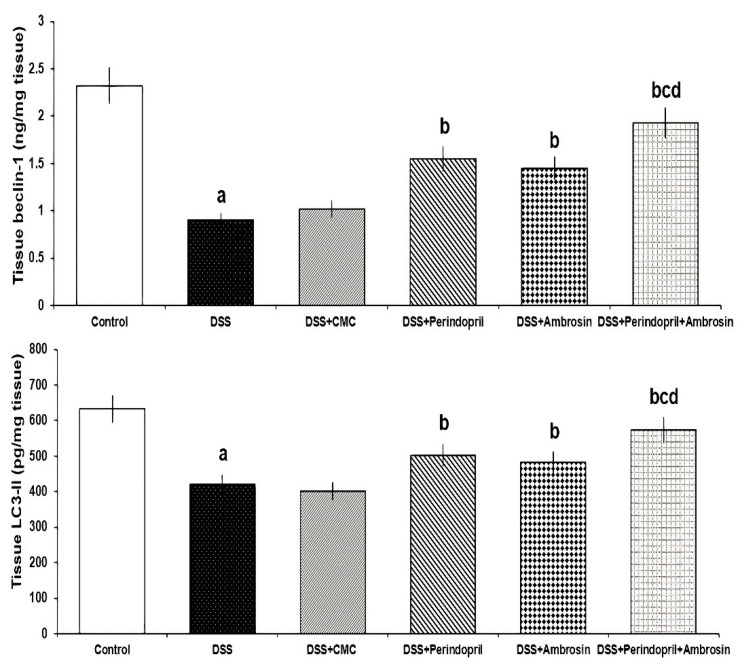
Effect of perindopril with or without ambrosin on beclin-1 and LC3-II levels in the colonic tissues of DSS-treated mice (Mean ± SD); where “a” denotes significant vs. the control group (*p*-value less than 0.05); “b” denotes significant vs. DSS group (*p*-value less than 0.05); “c” denotes significant vs. DSS + perindopril group (p-value less than 0.05); “d” denotes significant vs. DSS + ambrosin group (*p*-value less than 0.05).

Mice treated with either perindopril or ambrosin showed a significant increase in the levels of beclin-1 and LC3-II when put in comparison with mice treated with DSS, with the maximally encountered effect in mice treated with the perindopril/ambrosin combination. However, DSS mice treated with CMC did not exhibit any significant effect on beclin-1 and LC3-II levels relative to mice treated with DSS alone (Figure 3).

### 3.7. Effect of Perindopril and/or Ambrosin on Caspase-8 and Bcl-2 Levels in the Colonic Tissues of DSS-Treated Animals

**Figure 4:**
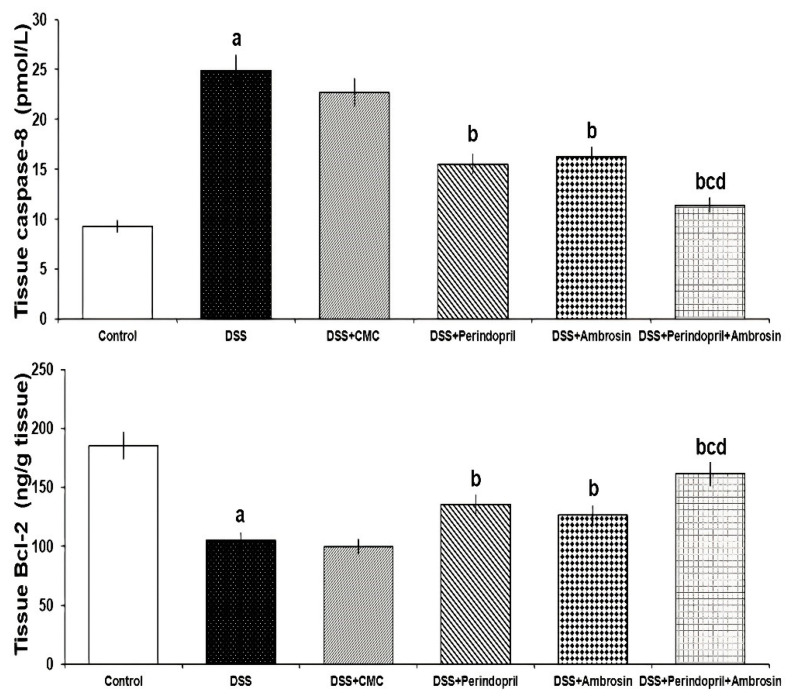
Effect of perindopril with or without ambrosin on caspase-8 and Bcl-2 levels in the colonic tissues of DSS-treated mice (Mean ± SD); where “a” denotes significant vs. the control group (*p*-value less than 0.05); “b” denotes significant vs. DSS group (*p*-value less than 0.05); “c” denotes significant vs. DSS + perindopril group (*p*-value less than 0.05); “d” denotes significant vs. DSS + ambrosin group (*p*-value less than 0.05).

Caspase-8 levels were elevated with the administration of DSS together with a significant decrement in Bcl-2 levels relative to the control untreated group. These effects were reversed in mice treated with perindopril with or without ambrosin, but DSS mice treated with the perindopril/ambrosin combination exhibited the most favorable response. However, CMC administered to DSS mice did not seem to have the ability to significantly affect either caspase-8 or Bcl-2 levels relative to mice treated with DSS alone (Figure 4).

### 3.8. Effect of Perindopril and/or Ambrosin on the Histopathological Changes and NF-κB (p65) Immunostaining in the Colonic Tissues of Mice Treated with DSS

**Figure 5:**
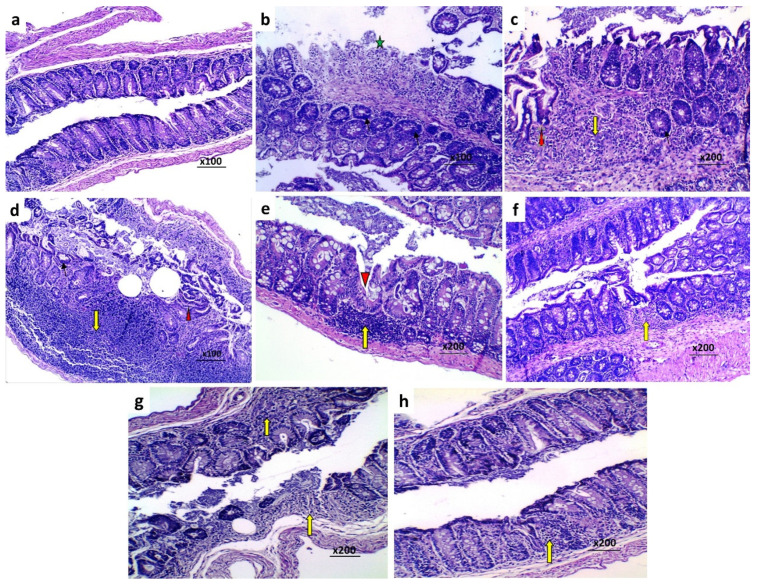
Hematoxylin and eosin stained sections of the colon of (**a**) The control untreated mice showing normal mucosa with intact mucosal glands, normal distribution of goblet cells and normal crypts (H and E ×100); (**b**) DSS mice showing surface ulceration of the colonic mucosa (Astrex) (×100); (**c**) DSS mice showing partial loss of the goblet cells (Thin arrow) with disruption of the crypts (Arrow head) (×200); (**d**) DSS mice showing massive inflammatory cellular infiltration mainly by lymphocytes (Thick arrow) (×400); (**e**) DSS mice treated with CMC showing disruption of the mucosal crypts (Arrow head) and inflammatory cellular infiltration mainly by lymphocytes (Arrow) (×200); (**f**) DSS mice treated with perindopril showing attenuated histological damage of the colonic tissues with minute ulcerations of the colonic mucosa and significant decrease in the inflammatory cellular infiltration (Arrow) (×200); (**g**) DSS mice treated with ambrosin showing significant amelioration of the histopathological damage of the colonic tissues with smaller ulcerations of the colonic mucosa and less dense inflammatory cellular infiltration (Arrows) (×200); (**h**) DSS mice treated with perindopril/ambrosin combination exhibiting restoration of the normal architecture of the colonic mucosa with absence of ulceration and mild inflammatory cellular infiltration (Arrow) (×200).

**Figure 6:**
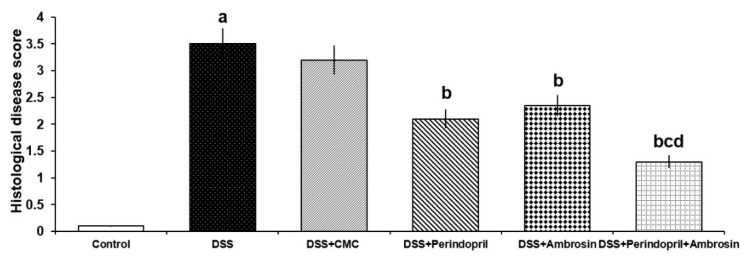
Effect of perindopril with or without ambrosin on the histological disease score in mice treated with DSS (Mean ± SD); where “a” denotes significant vs. the control group (*p*-value less than 0.05); “b” denotes significant vs. DSS group (*p*-value less than 0.05); “c” denotes significant vs. DSS + perindopril group (*p*-value less than 0.05); “d” denotes significant vs. DSS + ambrosin group (*p*-value less than 0.05).

**Figure 7:**
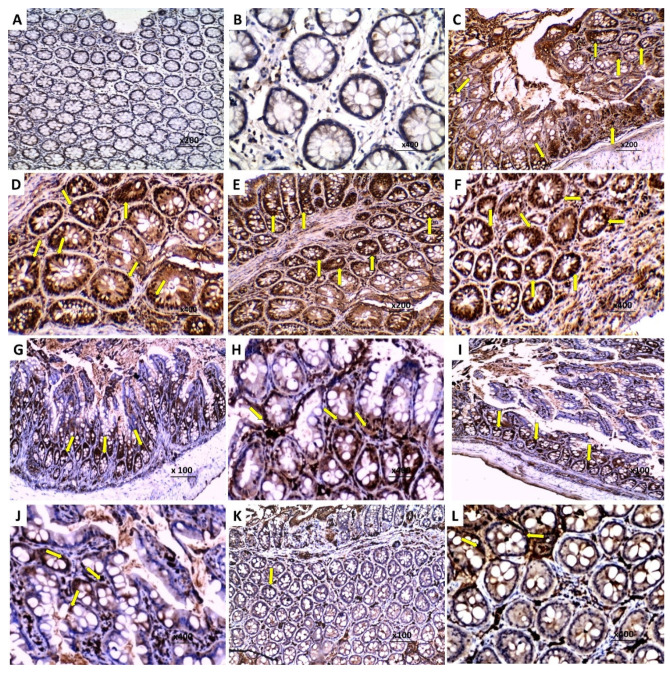
Photomicrographs of sections of the colon of (**A**,**B**) The control untreated mice showing negative nuclear immunoreactivity to NF-κB (p65) (×200 and ×400, respectively); (**C**,**D**) DSS mice exhibiting strong positive nuclear immunoreactivity to NF-κB (p65) in the colonic glands epithelial cells (Arrows) (×200 and ×400, respectively); (**E**,**F**) DSS mice treated with CMC showing strong positive nuclear immunoreactivity to NF-κB (p65) in the colonic glands epithelial cells (Arrows) (×200 and ×400, respectively); (**G**,**H**) DSS mice treated with perindopril exhibiting decreased immunoreactivity to NF-κB(p65) (moderate immunostaining) (Arrows) (×100 and ×400, respectively); (**I**,**J**) DSS mice treated with ambrosin showing decreased immunoreactivity to NF-κB (p65) (moderate immunostaining) (Arrows) (×100 and ×400, respectively); (**K**,**L**) DSS mice treated with perindopril/ambrosin combination revealing marked decrease in the immunoreactivity to NF-κB (p65) (mild immunostaining) (Arrow) (×100 and ×400, respectively).

The colonic tissues of mice treated with DSS alone showed surface epithelial ulceration, partial loss of goblet cells and intestinal crypts, and cellular inflammatory infiltration of the mucosa and submucosa mainly by lymphocytes (Figure 5b–d). Consequently, there was a significant increase in the histological disease score (Figure 6) and NF-κB (p65) immunostaining (Figure 7C,D) relative to the control untreated group. Daily administration of CMC to DSS-treated mice did not induce a significant change in the histopathological picture (Figure 5e), the histological disease score (Figure 6), or NF-κB (p65) immunostaining (Figure 7E,F) compared to mice treated with DSS alone. Each perindopril or ambrosin attenuated the histopathological tissue damage (Figure 5f,g) and induced a significant decrement in the histological disease score (Figure 6) and NF-κB (p65) immunostaining (Figure 7G–J) in the colonic tissues when compared to mice treated with DSS alone, but DSS mice treated with a perindopril/ambrosin combination exhibited the most favorable results (Figure 5h, Figure 6 and Figure 7K,L).

### 3.9. Perindopril and/or Ambrosin Combatted the Electron Microscopic Changes of the Colonic Tissues Induced by DSS Administration

**Figure 8:**
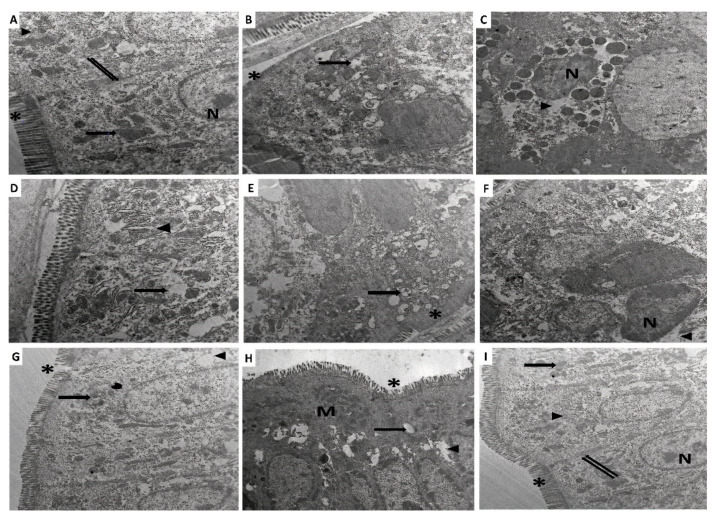
A transmission electron micrograph of a part of the colon of (**A**) The control untreated group showing absorptive columnar cells with apical microvilli (Asterisk), junctional complexes (Double arrows), mitochondria (Arrow), RER (Arrow head) and nuclei with prominent nucleoli (N) (×3000); (**B**) DSS mice exhibiting evident loss of the microvilli (Asterisk) and swollen mitochondria with destructed cristae (Arrow) (×3000); (**C**) DSS mice showing perinuclear cytoplasmic vacuolations (Arrow head) with hyperchromatic shrunken compressed nucleus (N) (×1500); (**D**) DSS mice exhibiting marked dilatation of RER (Arrow head) and marked cytoplasmic vacuolations (Arrow) (×3000); (**E**) DSS mice treated with CMC showing loss of the apical microvilli (Asterisk) and swollen mitochondria with destructed cristae (Arrow) (×2000); (**F**) DSS mice treated with CMC exhibiting perinuclear cytoplasmic vacuolations (Arrow head) and hyperchromatic shrunken compressed nucleus (N) (×3000); (**G**) DSS mice treated with perindopril showing partial loss of the apical microvilli (Asterisk), mild cytoplasmic vacuolation (Arrow head) and preserved normal mitochondria with scanty areas of mitochondrial swelling and destructed cristae (Arrow) (×2000); (**H**) DSS mice treated with ambrosin revealing nearly normal apical microvilli (Asterisk), cytoplasmic vacuolation (Arrow head) and preserved normal mitochondria (M) with scattered areas of mitochondrial swelling with destructed cristae (Arrow) (×2000); (**I**) DSS mice treated with perindopril/ambrosin combination showing apparently normal columnar cells with apical microvilli (Asterisk), RER (Arrow head), intercellular junctions (Double arrows), mitochondria (Arrow) and nucleus (N) (×2000).

In the control untreated group, the apical microvilli of the columnar cells were clearly determined, and the junctional complexes between the adjacent cells were normal. The cytoplasm of these cells showed normal mitochondria, rough endoplasmic reticulum (RER), and heterochromatic nuclei with prominent nucleoli (Figure 8A). Specimens obtained from animals treated with DSS alone showed marked cellular damage in the form of loss of the apical microvilli, extensive cytoplasmic and perinuclear vacuolations, mitochondrial swelling with the destruction of their cristae, dilated RER, and hyperchromatic compressed nucleus (Figure 8B–D). Administration of CMC to DSS-treated mice did not induce significant electron microscopic changes in the colonic tissues compared to mice treated with DSS alone (Figure 8E,F). Interestingly, mice treated with perindopril had partial loss of the apical microvilli, mild cytoplasmic vacuolation, and preserved normal mitochondria. However, scattered areas of mitochondrial swelling with destructed cristae were observed (Figure 8G). The ambrosin-treated group showed nearly normal apical microvilli and cytoplasmic vacuolation with mild mitochondrial swelling and partially destructed cristae (Figure 8H). Most of the normal architecture of the colon was restored in mice treated with perindopril/ambrosin in combination with apparently normal apical microvilli of the columnar cells with normal junctional complexes in-between these cells. Moreover, the cytoplasm of these cells contained apparently normal RER and mitochondria, together with heterochromatic nuclei with prominent nucleoli (Figure 8I).

## 4. Discussion

Ulcerative colitis is a chronic non-specific inflammatory disease primarily affecting the mucosa and submucosa of the colon [26]. The etiological factors underlying the incidence of this disorder are still not completely explored [27]. Nevertheless, alteration of the gut microflora, enhanced generation of pro-inflammatory cytokines, overproduction of ROS, and modulation of autophagy/apoptosis balance were suggested as potential contributing factors [28]. The effect of these factors on the progression of ulcerative colitis was clearly evident in the present study, where administration of DSS to mice induced an inflammatory state in the colonic microenvironment accompanied by increased production of ROS, with the net result of the significant shortening of the colon, a significant increase in DAI and the histological disease score, and various changes in the electron microscopic picture of the colonic tissues relative to the control untreated group. 

Nrf2 has been proven to play a key role in the pathophysiology of DSS-induced colitis [29]. Recent evidence stated that Nrf2 has the ability to control the expression of certain genes whose products represent the key elements in the detoxication of ROS and reactive nitrogen species in the colonic tissues [30]. In addition, increased Nrf2 content in the colonic tissues was noted to be associated with increased levels of the antioxidant enzymes in the colonic mucosa with subsequent combating of the deleterious events associated with oxidative stress [31]. Moreover, agents that enhance heme oxygenase-1 activity and increase Nrf2 levels in experimentally induced colitis were reported to reduce ROS production, possibly via suppression of NF-κB activity and inhibition of iNOS expression in the colonic tissues [30]. These effects were clearly noticed in the present study, where the significant decrease in Nrf2 content of the colonic tissues enhanced by DSS was associated with a significantly increased iNOS and ROS levels and a significant increase in NF-κB (p65) immunostaining, concomitantly with significant decline in the levels of the antioxidant enzymes when compared versus the control group.

In the present study, perindopril administration to DSS-treated mice was associated with a significant increase in the levels of Nrf2 and the antioxidant enzymes, together with a significant decrement in iNOS and ROS levels when compared to mice treated with DSS alone. This may be explained by the findings of Zhu et al. [32], who reported that among other ACE inhibitors, perindopril exerts outstanding antioxidant effects in various body tissues. Ancion et al. [33] attributed the antioxidant effects of perindopril to its ability to decrease angiotensin II levels, thereby reducing ROS production. Moreover, Arab et al. [34] stated that inhibition of ACE was associated with Nrf2 overexpression with subsequent restoration of the pro-oxidant/antioxidant balance in the colonic tissues. An interesting finding in the current study was that ambrosin exhibited effective antioxidant effects in the colonic tissues. This may be carried out via the ability of ambrosin to inhibit NF-κB-mediated ROS production with subsequent potentiation of the antioxidant defense mechanisms [18].

SIRT1/PPAR-γ signaling was suggested to play a fundamental role in the pathogenesis of DSS-induced colitis [35]. PPAR-γ was proven to be highly expressed in the colonic epithelial cells of healthy individuals, but this expression was found to be significantly diminished in patients with ulcerative colitis [36]. In addition, PPAR-γ was reported to be highly expressed in the immune cells, namely, macrophages, and hence may attain an important role in the pathogenesis of the colonic inflammation in cases of ulcerative colitis [37]. Recent studies have proven that PPAR-γ enhances the expression of SIRT1, which, in turn, deacetylates the p65 subunit of NF-κB, leading to marked inhibition of NF-κB-mediated pro-inflammatory cytokines production with subsequent amelioration of the inflammatory process in the colonic epithelial cells [38]. Moreover, suppression of NF-κB expression in the colonic tissues may be reflected by downregulated expression of TGF-β1, with subsequent reduction of intestinal fibrosis [39]. In addition, inhibition of NF-κB leads to inhibition of iNOS in the intestinal mucosa with a decreased generation of ROS and reactive nitrogen species with subsequent amelioration of oxidative stress in inflammatory bowel diseases [40]. This was clearly evident in the present study, where DSS administration induced a significant decrease in SIRT1/PPAR-γ signaling associated with a significant increase in NF-κB, TGF-β1, IL-1β, and IL-6 levels concomitantly with significant histopathological and electron microscopic changes in the colonic tissues compared to the control untreated group.

Coinciding with the results of the current study, the crosstalk between TLR4, NLRP3 inflammasome, and NF-κB signaling was proven to be a key regulator of the pathophysiology of DSS-induced colitis [41]. DSS administration enhances the binding of the priming inflammatory stimuli with TLR4, which, in turn, activates NF-κB with subsequent conversion of the inactive form of the NLRP3 inflammasome to the active form [42]. The activated NLRP3 inflammasome was proven to enhance the caspase-1-mediated conversion of pro-IL1β and to active IL-1β that stimulates the production of IL-6 and the other proinflammatory cytokines with subsequent augmentation of the inflammatory processes in the colonic tissues [43]. Moreover, activation of the NLRP3 inflammasome enhances pyroptosis, an inflammatory form of cell death in the colonic mucosa [12]. This, in addition to the inhibitory effect of TLR4 on SIRT1 levels and its proven role in ROS production, supports the hypothesis that TLR4/NF-κB/NLRP3 inflammasome signaling is the keystone in the inflammatory events encountered in DSS-induced colitis [13].

The c-Fos and c-Jun are proto-oncogenes that encode certain proteins that regulate the transcription of promoters containing activator protein-1 (AP-1) elements [44]. In DSS-induced colitis, activation of MAPKs may lead to enhanced expression of c-Fos and c-Jun with a subsequent increase in the production of AP-1 complexes, which together with NF-κB promote the expression of a wide range of the pro-inflammatory mediators involved in colonic inflammation [45]. This was in the same line with the data obtained from the current work where DSS administration induced significant elevation of phosphorylated p38 MAPK, c-Fos, and c-Jun with subsequent activation of the inflammatory processes in the colonic tissues, as evidenced by the biochemical, histopathological, and electron microscopic findings.

The current work focused light on the potential effect of perindopril on the inflammatory events that occur in the colonic mucosa in DSS-induced colitis. Coinciding with the results of the present work, Sayed et al. [38] suggested that ACE inhibition might decrease the production of the proinflammatory cytokines in the colonic tissues via enhancing the SIRT1/PPAR-γ signaling pathways. Jaworska et al. [46] established a relationship between the tissue levels of angiotensin II and the expression of TLR4 in the colonic tissues. In addition, Sayed et al. [38] reported that ACE inhibitors have the ability to suppress the activity of the p65 subunit of NF-κB with subsequent mitigation of NLRP3 inflammasome expression, leading to significant amelioration of the inflammatory processes in the colonic tissues. Moreover, agents that affect the angiotensin II levels were reported to modulate the MAPK/c-Fos/c-Jun signaling pathway with subsequent combatting of the inflammatory events induced by DSS in the colonic tissues [47]. An interesting finding in the present study was that ambrosin exerted potent anti-inflammatory effects in the colonic tissues of mice treated with DSS. This was in accordance with the findings of Khalil et al. [18], who attributed these effects to the ability of ambrosin to decrease the levels of NF-κB to such a great extent that it significantly affects the signaling pathways in which NF-κB might play a role.

Data obtained from the present study were in agreement with the accumulated evidence that there is a strong relationship between autophagy and DSS-induced colitis [48]. Autophagy in the intestinal epithelium represents a key element in the maintenance of intestinal homeostasis, possibly via augmentation of the immunological functions of Paneth cells in the intestine [49]. Autophagy deficiency has been reported to exacerbate DSS-induced colitis, possibly through exacerbation of the deleterious events resulting from oxidative stress and through activation of the MAPK signaling pathway [50]. Accumulation of the autophagy protein LC3-II was proven to represent an effective mechanism by which agents that augment autophagy may combat the deleterious effects exerted by DSS on the intestinal mucosa [51]. 

The findings of the current work have proven that augmentation of the mediators of autophagy may represent a vital mechanism by which perindopril might combat the pathogenic effects exerted by DSS on the colonic epithelium. Zhang et al. [52] reported that agents affecting ACE are capable of increasing beclin-1 levels, which, in turn, leads to accumulation of LC3-II with subsequent augmentation of autophagy. Moreover, enhancement of the signaling pathways related to autophagy induced by ambrosin administration in the present study may be an indirect consequence of the significant inhibition of the p65 subunit of NF-κB with the net result of increased beclin-1 levels [18,53].

Apoptosis represents a well-proven mechanism by which DSS may affect the colonic mucosa, leading to ulcerative colitis [54]. Coinciding with the data obtained from the current work, DSS administration was reported an increase caspase-8 levels, which, consequently, enhances TNF-α induced an epithelial necroptosis and promotes the development of colitis [55]. Agents that had the ability to modulate caspase-8 expression in the colonic tissues not only combated the apoptotic pathways but also exhibited potent anti-inflammatory effects [56]. In addition, DSS was reported to negatively influence the levels of the antiapoptotic proteins, including Bcl-2, with resultant augmentation of apoptosis in the colonic tissues [57]. These apoptosis-inducing properties of DSS were significantly combated in the present study in mice treated with perindopril, an interesting finding which complied with the results of Yang et al. [58]. They attributed the antiapoptotic effects of perindopril to its ability to combat the genetic expression of caspases together with upregulation of Bcl-2 that interferes with apoptosis at the different cellular levels. In addition, targeting the NF-κB/Bcl2 signaling pathway by ambrosin might be an acceptable explanation for the apoptosis-modulating effects of ambrosin that were observed in the present study [18,59].

**Figure 9:**
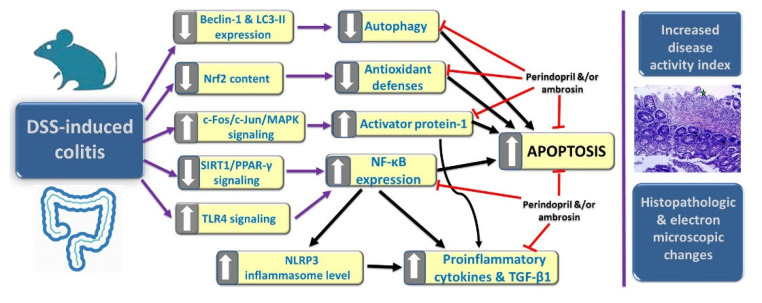
A summative diagram showing the mechanisms, by which perindopril and/or ambrosin may combat DSS-induced colitis.

In the current study, administration of the perindopril/ambrosin combination to DSS-treated mice elicited a significant decrease in DAI, restoration of the pro-oxidant/antioxidant balance, amelioration of the inflammatory signaling pathways, augmentation of the signaling pathways related to autophagy, together with inhibition of apoptosis relative to DSS mice treated with perindopril or ambrosin alone. This may be attributed, to a large extent, to the synergistic antioxidant and anti-inflammatory effects of both agents, in addition to their ability to restore autophagy/apoptosis balance (Figure 9). Moreover, the well-proven antibacterial effects of ambrosin against intestinal pathogens may give suitable circumstances for the growth of the normal colonic microflora that were proven to have a fundamental role in the innate immunity against the development of ulcerative colitis [60]. 

## 5. Conclusions

The perindopril/ambrosin combination might represent an effective modality for mitigation of the pathogenic events and the clinical sequelae of colitis. Supplemental investigations are needed to identify the exact molecular mechanisms that underlie the effects of perindopril with or without ambrosin on DSS-induced colitis, including cellular and subcellular localization of the target proteins.

## Figures and Tables

**Table 1:** The scoring system for the disease activity index.

Score	Weight Loss (%)	Stool Consistency	Hematochezia
0	None	Normal	Normal
1	0–10	-------	-------
2	11–15	Loose	Hemoccult positive
3	16–20	-------	-------
4	>20	Diarrhea	Gross bleeding

**Table 2:** Effect of perindopril with or without ambrosin on the antioxidant enzymes, ROS, and iNOS/Nrf2 signaling in the colonic tissues of DSS-treated mice (Mean ± SD).

Parameters	Control (*n* = 8)	DSS (*n* = 8)	DSS + CMC (*n* = 8)	DSS + Perindopril (*n* = 8)	DSS + Ambrosin (*n* = 8)	DSS + Perindopril + Ambrosin (*n* = 8)
Tissue glutathione reductase (U/g tissue)	152.25 ± 18.33	53.41 ± 7.89 ^a^	49.94 ± 7.19	93.41 ± 11.72 ^b^	86.27 ± 9.83 ^b^	126.84 ± 14.3 ^bcd^
Tissue glutathione peroxidase (U/g tissue)	35.31 ± 4.71	13.72 ± 1.85 ^a^	14.37 ± 1.89	24.13 ± 2.93 ^b^	21.81 ± 2.68 ^b^	30.39 ± 4.03 ^bcd^
Tissue ROS (U/g tissue)	56.47 ± 6.23	174.86 ± 19.27 ^a^	167.3 ± 18.51	98.46 ± 10.75 ^b^	112.81 ± 13.02 ^b^	74.37 ± 8.24 ^bcd^
Tissue iNOS (ng/g tissue)	21.46 ± 2.82	45.57 ± 5.61 ^a^	48.19 ± 5.92	33.67 ± 3.95 ^b^	38.56 ± 4.23 ^b^	26.76 ± 3.1 ^bcd^
Tissue Nrf2 content (×10^−1^ ng/mg protein)	0.47 ± 0.05	0.21 ± 0.03 ^a^	0.23 ± 0.03	0.34 ± 0.04 ^b^	0.31 ± 0.04 ^b^	0.41 ± 0.05 ^bcd^

^a^ Significant vs. the control group (*p*-value less than 0.05); ^b^ Significant vs. DSS group (*p*-value less than 0.05); ^c^ Significant vs. DSS + perindopril group (*p*-value less than 0.05); ^d^ Significant vs. DSS + ambrosin group (*p*-value less than 0.05). DSS: dextran sulphate sodium; CMC: carboxymethyl cellulose.

**Table 3:** Effect of perindopril with or without ambrosin on TLR4, IL-1β, IL-6, and TGF-β1 in the colonic tissues of DSS-treated mice (Mean ± SD).

Parameters	Control (*n* = 8)	DSS (*n* = 8)	DSS + CMC (*n* = 8)	DSS + Perindopril (*n* = 8)	DSS + Ambrosin (*n* = 8)	DSS + Perindopril + Ambrosin (*n* = 8)
Tissue TLR-4 (ng/g tissue)	321.47 ± 42.3	797.5 ± 87.13 ^a^	776.3 ± 82.17	547.7 ± 62.79 ^b^	595.3 ± 68.18 ^b^	427.8 ± 52.61 ^bcd^
Tissue IL-1β (pg/mg protein)	477.3 ± 54.5	1275.8 ± 146.7 ^a^	1298.3 ± 155.6	883.5 ± 97.4 ^b^	973.2 ± 112.36 ^b^	643.2 ± 75.23 ^bcd^
Tissue IL-6 (pg/mg protein)	231.64 ± 33.28	686.12 ± 75.76 ^a^	662.96 ± 72.57	432.49 ± 51.7 ^b^	474.38 ± 56.27 ^b^	331.67 ± 43.76 ^bcd^
Tissue TGF-β1 (pg/mg protein)	19.35 ± 2.93	78.38 ± 8.31 ^a^	83.1 ± 8.83	48.53 ± 5.64 ^b^	55.26 ± 6.23 ^b^	33.78 ± 4.52 ^bcd^

^a^ Significant vs. the control group (p-value less than 0.05); ^b^ Significant vs. DSS group (*p*-value less than 0.05); ^c^ Significant vs. DSS + perindopril group (*p*-value less than 0.05); ^d^ Significant vs. DSS + ambrosin group (*p*-value less than 0.05). DSS: dextran sulphate sodium; CMC: carboxymethyl cellulose.

**Table 4:** Effect of perindopril with or without ambrosin on phospho-p38 MAPK, c-Fos, c-Jun, and NLRP3 inflammasome in the colonic tissues of dextran sulphate sodium (DSS)-treated mice (Mean ± SD).

Parameters	Control (*n* = 8)	DSS (*n* = 8)	DSS + CMC (*n* = 8)	DSS + Perindopril (*n* = 8)	DSS + Ambrosin (*n* = 8)	DSS + Perindopril + Ambrosin (*n* = 8)
Tissue phospho-p38 MAPK (% change from the control)	100.00 ± 12.11	178.65 ± 19.34 ^a^	183.94 ± 19.88	141.75 ± 15.84 ^b^	149.45 ± 16.15 ^b^	123.17 ± 14.93 ^bcd^
Tissue c-Fos (% change from the control)	100.00 ± 9.23	229.75 ± 25.73 ^a^	234.24 ± 28.16	173.41 ± 19.8 ^b^	188.74 ± 21.03 ^b^	144.72 ± 17.36 ^bcd^
Tissue c-Jun (% change from the control)	100.00 ± 11.65	257.36 ± 29.45 ^a^	249.72 ± 28.15	181.43 ± 20.47 ^b^	196.74 ± 21.42 ^b^	153.78 ± 17.79 ^bcd^
Tissue NLRP3 inflammasome (pg/mg protein)	186.3 ± 20.45	572.5 ± 63.48 ^a^	589.7 ± 68.33	364.5 ± 41.76 ^b^	391.92 ± 45.8 ^b^	277.56 ± 34.8 ^bcd^

^a^ Significant vs. the control group (*p*-value less than 0.05); ^b^ Significant vs. DSS group (*p*-value less than 0.05); ^c^ Significant vs. DSS + perindopril group (*p*-value less than 0.05); ^d^ Significant vs. DSS + ambrosin group (*p*-value less than 0.05). DSS: dextran sulphate sodium; CMC: carboxymethyl cellulose.

## Data Availability

Data is contained within the article.

## References

[1] Porter R.J., Kalla R., Ho G.T. (2020). Ulcerative colitis: Recent advances in the understanding of disease pathogenesis. F1000Research.

[2] Passos M.A.T., Chaves F.C., Chaves-Junior N. (2018). The Importance of Colonoscopy in Inflammatory Bowel Diseases. Arq. Bras. Cir. Dig..

[3] Kaur A., Goggolidou P. (2020). Ulcerative colitis: Understanding its cellular pathology could provide insights into novel therapies. J. Inflamm..

[4] Gatti S., Gelzoni G., Catassi G.N., Catassi C. (2021). The Clinical Spectrum of Inflammatory Bowel Disease Associated with Specific Genetic Syndromes: Two Novel Pediatric Cases and a Systematic Review. Front. Pediatr..

[5] Malik A., Stringer E., Warner N., van Limbergen J., Vandersteen A., Muise A., Derfalvi B. (2021). Multisystem Autoimmune Inflammatory Disease, Including Colitis, Due to Inborn Error of Immunity. Pediatrics.

[6] Yu Y.R., Rodriguez J.R. (2017). Clinical presentation of Crohn’s, ulcerative colitis, and indeterminate colitis: Symptoms, extraintestinal manifestations, and disease phenotypes. Semin. Pediatr. Surg..

[7] Negreanu L., Voiosu T., State M., Voiosu A., Bengus A., Mateescu B.R. (2019). Endoscopy in inflammatory bowel disease: From guidelines to real life. Therap. Adv. Gastroenterol..

[8] Ghosh S., Sanchez Gonzalez Y., Zhou W., Clark R., Xie W., Louis E., Loftus E.V., Panes J., Danese S. (2021). Upadacitinib Treatment Improves Symptoms of Bowel Urgency and Abdominal Pain and Correlates with Quality of Life Improvements in Patients with Moderate to Severe Ulcerative Colitis. J. Crohns Colitis.

[9] Tatiya-Aphiradee N., Chatuphonprasert W., Jarukamjorn K. (2018). Immune response and inflammatory pathway of ulcerative colitis. J. Basic Clin. Physiol. Pharmacol..

[10] Mohammad Jafari R., Shayesteh S., Ala M., Yousefi-Manesh H., Rashidian A., Hashemian S.M., Sorouri M., Dehpour A.R. (2021). Dapsone Ameliorates Colitis through TLR4/NF-kB Pathway in TNBS Induced Colitis Model in Rat. Arch. Med. Res..

[11] He X., Wei Z., Wang J., Kou J., Liu W., Fu Y., Yang Z. (2016). Alpinetin attenuates inflammatory responses by suppressing TLR4 and NLRP3 signaling pathways in DSS-induced acute colitis. Sci. Rep..

[12] Wu X., Pan S., Luo W., Shen Z., Meng X., Xiao M., Tan B., Nie K., Tong T., Wang X. (2020). Roseburia intestinalis-derived flagellin ameliorates colitis by targeting miR-223-3p-mediated activation of NLRP3 inflammasome and pyroptosis. Mol. Med. Rep..

[13] Abdel-Wahab B.A., Alkahtani S.A., Alqahtani A.A., Hassanein E.H.M. (2022). Umbelliferone ameliorates ulcerative colitis induced by acetic acid via modulation of TLR4/NF-κB-p65/iNOS and SIRT1/PPARγ signaling pathways in rats. Environ. Sci. Pollut. Res. Int..

[14] Ferreira-Duarte M., Rodrigues-Pinto T., Sousa T., Faria M.A., Rocha M.S., Menezes-Pinto D., Esteves-Monteiro M., Magro F., Dias-Pereira P., Duarte-Araújo M. (2021). Interaction between the Renin-Angiotensin System and Enteric Neurotransmission Contributes to Colonic Dysmotility in the TNBS-Induced Model of Colitis. Int. J. Mol. Sci..

[15] Jacobs J.D., Wagner T., Gulotta G., Liao C., Li Y.C., Bissonnette M., Pekow J. (2019). Impact of Angiotensin II Signaling Blockade on Clinical Outcomes in Patients with Inflammatory Bowel Disease. Dig. Dis. Sci..

[16] Santiago O.I., Rivera E., Ferder L., Appleyard C.B. (2008). An angiotensin II receptor antagonist reduces inflammatory parameters in two models of colitis. Regul. Pept..

[17] Svensson D., Lozano M., Almanza G.R., Nilsson B.O., Sterner O., Villagomez R. (2018). Sesquiterpene lactones from Ambrosia arborescens Mill. inhibit pro-inflammatory cytokine expression and modulate NF-κB signaling in human skin cells. Phytomedicine.

[18] Khalil M.N.A., Choucry M.A., El Senousy A.S., Hassan A., El-Marasy S.A., El Awdan S.A., Omar F.A. (2019). Ambrosin, a potent NF-κβ inhibitor, ameliorates lipopolysaccharide induced memory impairment, comparison to curcumin. PLoS ONE.

[19] Arya A., Chahal R., Rao R., Rahman M.H., Kaushik D., Akhtar M.F., Saleem A., Khalifa S.M.A., El-Seedi H.R., Kamel M. (2021). Acetylcholinesterase Inhibitory Potential of Various Sesquiterpene Analogues for Alzheimer′s Disease Therapy. Biomolecules.

[20] Abouzid S., Elshahaat A., Ali S., Choudhary M.I. (2008). Antioxidant activity of wild plants collected in Beni-Sueif governorate, Upper Egypt. Drug Discov. Ther..

[21] Dong J., Chen Y., Yang F., Zhang W., Wei K., Xiong Y., Wang L., Zhou Z., Li C., Wang J. (2021). Naringin Exerts Therapeutic Effects on Mice Colitis: A Study Based on Transcriptomics Combined with Functional Experiments. Front. Pharmacol..

[22] Yamada K., Uchida S., Takahashi S., Takayama M., Nagata Y., Suzuki N., Shirakura S., Kanda T. (2010). Effect of a centrally active angiotensin-converting enzyme inhibitor, perindopril, on cognitive performance in a mouse model of Alzheimer′s disease. Brain Res..

[23] Friedman D.J., Künzli B.M., A-Rahim Y.I., Sevigny J., Berberat P.O., Enjyoji K., Csizmadia E., Friess H., Robson S.C. (2009). From the Cover: CD39 deletion exacerbates experimental murine colitis and human polymorphisms increase susceptibility to inflammatory bowel disease. Proc. Natl. Acad. Sci. USA.

[24] Hirata I., Yasumoto S., Toshina K., Inoue T., Nishikawa T., Murano N., Murano M., Wang F.Y., Katsu K. (2007). Evaluation of the effect of pyrrolidine dithiocarbamate in suppressing inflammation in mice with dextran sodium sulfate-induced colitis. World J. Gastroenterol..

[25] Andresen L., Jørgensen V.L., Perner A., Hansen A., Eugen-Olsen J., Rask-Madsen J. (2005). Activation of nuclear factor KappaB in colonic mucosa from patients with collagenous and ulcerative colitis. Gut.

[26] Ungaro R., Mehandru S., Allen P.B., Peyrin-Biroulet L., Colombel J.F. (2017). Ulcerative colitis. Lancet.

[27] Kobayashi T., Siegmund B., Le Berre C., Wei S.C., Ferrante M., Shen B., Bernstein C.N., Danese S., Peyrin-Biroulet L., Hibi T. (2020). Ulcerative colitis. Nat. Rev. Dis. Primers..

[28] Samoilă I., Dinescu S., Costache M. (2020). Interplay between Cellular and Molecular Mechanisms Underlying Inflammatory Bowel Diseases Development-A Focus on Ulcerative Colitis. Cells.

[29] Wang R., Wang D., Wang H., Wang T., Weng Y., Zhang Y., Luo Y., Lu Y., Wang Y. (2021). Therapeutic Targeting of Nrf2 Signaling by Maggot Extracts Ameliorates Inflammation-Associated Intestinal Fibrosis in Chronic DSS-Induced Colitis. Front. Immunol..

[30] Pompili S., Sferra R., Gaudio E., Viscido A., Frieri G., Vetuschi A., Latella G. (2019). Can Nrf2 Modulate the Development of Intestinal Fibrosis and Cancer in Inflammatory Bowel Disease?. Int. J. Mol. Sci..

[31] Liu H., Johnston L.J., Wang F., Ma X. (2021). Triggers for the Nrf2/ARE Signaling Pathway and Its Nutritional Regulation: Potential Therapeutic Applications of Ulcerative Colitis. Int. J. Mol. Sci..

[32] Zhu Z., Li H., Chen W., Cui Y., Huang A., Qi X. (2020). Perindopril Improves Cardiac Function by Enhancing the Expression of SIRT3 and PGC-1α in a Rat Model of Isoproterenol-Induced Cardiomyopathy. Front. Pharmacol..

[33] Ancion A., Tridetti J., Nguyen Trung M.L., Oury C., Lancellotti P. (2019). A Review of the Role of Bradykinin and Nitric Oxide in the Cardioprotective Action of Angiotensin-Converting Enzyme Inhibitors: Focus on Perindopril. Cardiol. Ther..

[34] Arab H.H., Al-Shorbagy M.Y., Abdallah D.M., Nassar N.N. (2014). Telmisartan attenuates colon inflammation, oxidative perturbations and apoptosis in a rat model of experimental inflammatory bowel disease. PLoS ONE.

[35] Guo C., Zhang Y., Ling T., Zhao C., Li Y., Geng M., Gai S., Qi W., Luo X., Chen L. (2022). Chitosan Oligosaccharides Alleviate Colitis by Regulating Intestinal Microbiota and PPARγ/SIRT1-Mediated NF-κB Pathway. Mar. Drugs.

[36] Decara J., Rivera P., López-Gambero A.J., Serrano A., Pavón F.J., Baixeras E., Rodríguez de Fonseca F., Suárez J. (2020). Peroxisome Proliferator-Activated Receptors: Experimental Targeting for the Treatment of Inflammatory Bowel Diseases. Front. Pharmacol..

[37] Dou X., Xiao J., Jin Z., Zheng P. (2015). Peroxisome proliferator-activated receptor-γ is downregulated in ulcerative colitis and is involved in experimental colitis-associated neoplasia. Oncol. Lett..

[38] Sayed A.M., Abdel-Fattah M.M., Arab H.H., Mohamed W.R., Hassanein E.H.M. (2022). Targeting inflammation and redox aberrations by perindopril attenuates methotrexate-induced intestinal injury in rats: Role of TLR4/NF-κB and c-Fos/c-Jun pro-inflammatory pathways and PPAR-γ/SIRT1 cytoprotective signals. Chem. Biol. Interact..

[39] Curciarello R., Docena G.H., MacDonald T.T. (2017). The Role of Cytokines in the Fibrotic Responses in Crohn’s Disease. Front. Med..

[40] Tian T., Wang Z., Zhang J. (2017). Pathomechanisms of Oxidative Stress in Inflammatory Bowel Disease and Potential Antioxidant Therapies. Oxid. Med. Cell Longev..

[41] Chen Y., Lu Y., Pei C., Liang J., Ding P., Chen S., Hou S.Z. (2020). Monotropein alleviates secondary liver injury in chronic colitis by regulating TLR4/NF-κB signaling and NLRP3 inflammasome. Eur. J. Pharmacol..

[42] Wagatsuma K., Nakase H. (2020). Contradictory Effects of NLRP3 Inflammasome Regulatory Mechanisms in Colitis. Int. J. Mol. Sci..

[43] Zhen Y., Zhang H. (2019). NLRP3 Inflammasome and Inflammatory Bowel Disease. Front. Immunol..

[44] Langfermann D.S., Rössler O.G., Thiel G. (2018). Stimulation of B-Raf increases c-Jun and c-Fos expression and upregulates AP-1-regulated gene transcription in insulinoma cells. Mol. Cell Endocrinol..

[45] Lertnimitphun P., Jiang Y., Kim N., Fu W., Zheng C., Tan H., Zhou H., Zhang X., Pei W., Lu Y. (2019). Safranal Alleviates Dextran Sulfate Sodium-Induced Colitis and Suppresses Macrophage-Mediated Inflammation. Front. Pharmacol..

[46] Jaworska K., Koper M., Ufnal M. (2021). Gut microbiota and renin-angiotensin system: A complex interplay at local and systemic levels. Am. J. Physiol. Gastrointest. Liver Physiol..

[47] Broom O.J., Widjaya B., Troelsen J., Olsen J., Nielsen O.H. (2009). Mitogen activated protein kinases: A role in inflammatory bowel disease?. Clin. Exp. Immunol..

[48] Shao B.Z., Yao Y., Zhai J.S., Zhu J.H., Li J.P., Wu K. (2021). The Role of Autophagy in Inflammatory Bowel Disease. Front. Physiol..

[49] Haq S., Grondin J., Banskota S., Khan W.I. (2019). Autophagy: Roles in intestinal mucosal homeostasis and inflammation. J. Biomed. Sci..

[50] Kubota M., Kakimoto K., Nakagawa T., Koubayashi E., Nakazawa K., Tawa H., Hirata Y., Okada T., Kawakami K., Asai A. (2019). Autophagy deficiency exacerbates colitis through excessive oxidative stress and MAPK signaling pathway activation. PLoS ONE.

[51] Xie J., Li L., Deng S., Chen J., Gu Q., Su H., Wen L., Wang S., Lin C., Qi C. (2020). Slit2/Robo1 Mitigates DSS-induced Ulcerative Colitis by Activating Autophagy in Intestinal Stem Cell. Int. J. Biol. Sci..

[52] Zhang X., Zheng J., Yan Y., Ruan Z., Su Y., Wang J., Huang H., Zhang Y., Wang W., Gao J. (2019). Angiotensin-converting enzyme 2 regulates autophagy in acute lung injury through AMPK/mTOR signaling. Arch. Biochem. Biophys..

[53] Lim C.B., Fu P.Y., Ky N., Zhu H.S., Feng X., Li J., Srinivasan K.G., Hamza M.S., Zhao Y. (2012). NF-κB p65 repression by the sesquiterpene lactone, Helenalin, contributes to the induction of autophagy cell death. BMC Complement. Altern. Med..

[54] Shen N., Wang Z., Wang C., Zhang J., Liu C. (2020). Methane Alleviates Inflammation and Apoptosis of Dextran Sulfate Sodium-Induced Inflammatory Bowel Diseases by Inhibiting Toll-Like Receptor 4 (TLR4)/Myeloid Differentiation Factor 88 (MyD88)/Nuclear Translocation of Nuclear Factor-κB (NF-κB) and Endoplasmic Reticulum Stress Pathways in Mice. Med. Sci. Monit..

[55] Günther C., Martini E., Wittkopf N., Amann K., Weigmann B., Neumann H., Waldner M.J., Hedrick S.M., Tenzer S., Neurath M.F. (2011). Caspase-8 regulates TNF-α-induced epithelial necroptosis and terminal ileitis. Nature.

[56] Tummers B., Green D.R. (2017). Caspase-8: Regulating life and death. Immunol. Rev..

[57] Diarra A., Eissa N., Ghia J. (2020). A212 Chromofungin Protects Against DSS-Induced Colitis by Regulating P-53 Apoptitic Pathway. J. Can. Assoc. Gastroenterol..

[58] Yang W., Shi L., Chen L., Zhang B., Ma K., Liu Y., Qian Y. (2014). Protective effects of perindopril on d-galactose and aluminum trichloride induced neurotoxicity via the apoptosis of mitochondria-mediated intrinsic pathway in the hippocampus of mice. Brain Res. Bull..

[59] Fan S., Cui Y., Hu Z., Wang W., Jiang W., Xu H. (2020). Ambrosin sesquiterpene lactone exerts selective and potent anticancer effects in drug-resistant human breast cancer cells (MDA-MB-231) through mitochondrial mediated apoptosis, ROS generation and targeting Akt/β-Catenin signaling pathway. J. Buon..

[60] Wang P., Kong C.H., Zhang C.X. (2006). Chemical composition and antimicrobial activity of the essential oil from *Ambrosia trifida* L.. Molecules.

